# CD138 expression is a molecular signature but not a developmental requirement for ROR**γ**t^+^ NKT17 cells

**DOI:** 10.1172/jci.insight.148038

**Published:** 2021-09-22

**Authors:** Shunqun Luo, Juntae Kwon, Assiatu Crossman, Pyong Woo Park, Jung-Hyun Park

**Affiliations:** 1 Experimental Immunology Branch, Center for Cancer Research, National Cancer Institute, NIH, Bethesda, Maryland, USA.; 2 Department of Medicine, Boston Children’s Hospital, Harvard Medical School, Boston, Massachusetts, USA.

**Keywords:** Immunology, NKT cells, T cell development, T cells

## Abstract

Invariant NKT (*i*NKT) cells are potent immunomodulatory cells that acquire effector function during their development in the thymus. IL-17–producing *i*NKT cells are commonly referred to as NKT17 cells, and they are unique among *i*NKT cells to express the heparan sulfate proteoglycan CD138 and the transcription factor RORγt. Whether and how CD138 and RORγt contribute to NKT17 cell differentiation, and whether there is an interplay between RORγt and CD138 expression to control *i*NKT lineage fate, remain mostly unknown. Here, we showed that CD138 expression was only associated with and not required for the differentiation and IL-17 production of NKT17 cells. Consequently, CD138-deficient mice still generated robust numbers of IL-17–producing RORγt^+^ NKT17 cells. Moreover, forced expression of RORγt significantly promoted the generation of thymic NKT17 cells, but did not induce CD138 expression on non-NKT17 cells. These results indicated that NKT17 cell generation and IL-17 production were driven by RORγt, employing mechanisms that were independent of CD138. Therefore, our study effectively dissociated CD138 expression from the RORγt-driven molecular pathway of NKT17 cell differentiation.

## Graphical abstract

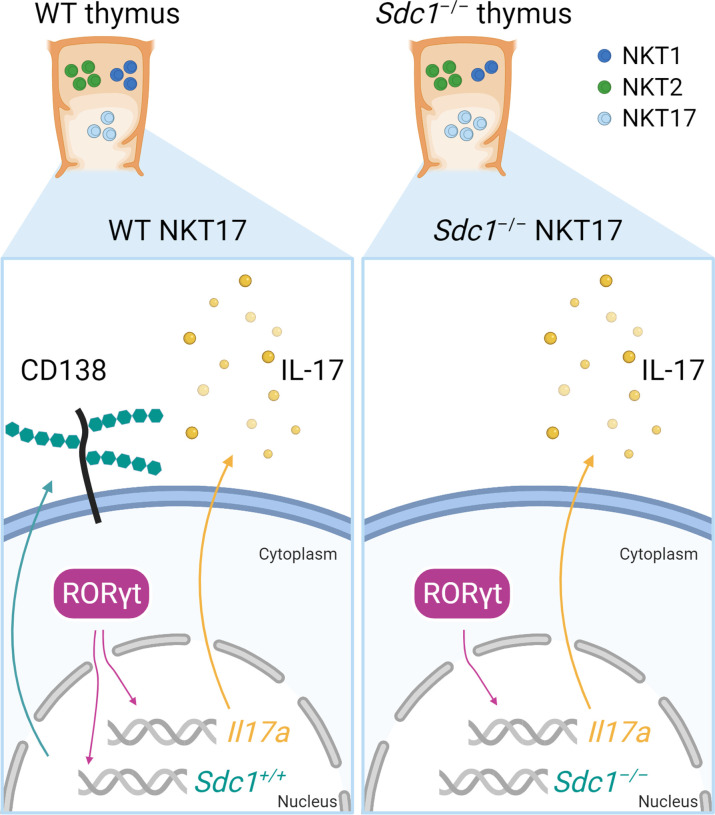

## Introduction

Invariant NKT (*i*NKT) cells are generated from immature CD4^+^CD8^+^ double-positive thymocytes upon their positive selection by glycolipid-loaded CD1d molecules (1, 2). Most *i*NKT cells share an invariant Vα14-Jα18 TCRα chain, resulting in limited antigen specificity, but they are still diverse in their effector function and cytokine expression (3). Three major subsets of *i*NKT cells arise in the thymus, and they are commonly referred to as NKT1, NKT2, and NKT17 cells based on their signature transcription factor and cytokine expression profiles (4–6). Analogous to the T helper subsets in CD4 effector T cells, *i*NKT cells that express the transcription factor T-bet and produce the cytokine IFN-γ are known as NKT1 cells. On the other hand, *i*NKT cells that express the transcription factor RORγt and produce IL-17 are referred to as NKT17 cells (4, 6). Thus, T-bet and RORγt are distinctly expressed in NKT1 and NKT17 cells as is the case for Th1 and Th17 CD4 helper T cells, respectively. The IL-4–producing *i*NKT subset is commonly referred to as NKT2, and NKT2 cells are primarily identified by the expression of large amounts of the zinc finger protein PLZF (4, 7, 8). In fact, high-level expression of PLZF is a more stringent marker for NKT2 cells than the expression of the conventional Th2-lineage marker GATA3 because GATA3 is promiscuously expressed among *i*NKT subsets (9). Collectively, the selective expression of key transcription factors is associated with and identifies individual *i*NKT subsets. However, it remains unclear how such *i*NKT subset identity is established during development in the thymus (10).

For NKT1 cells, the cytokine receptor CD122 (IL-2Rβ) is critical for their generation, most likely because CD122 is required for IL-15 signaling, which in turn induces the expression of T-bet, the master transcription factor of NKT1 cells (11, 12). Because CD122 is exclusively expressed on NKT1 cells (4), these data further suggest that CD122 expression is both required for and associated with NKT1 subset differentiation (13). Moreover, T-bet upregulates the expression of CD122 (14), driving a self-reinforcing circuitry for NKT1 cell differentiation. Accordingly, CD122 signaling induces T-bet to impose NKT1 lineage fate and to upregulate CD122 expression, which then results in increased IL-15 signaling and further increases the amount of T-bet expression.

In NKT17 cells, a cell-surface heparan sulfate proteoglycan, i.e., CD138 (syndecan-1), was recently identified as a subset-specific molecule exclusively found in NKT17 cells (15). It is conceivable that CD138 could play a similar role to CD122 in driving *i*NKT subset differentiation, with CD138 expression both associated with and required for NKT17 cell generation. However, the developmental pathway of thymic NKT17 cell generation remains incompletely mapped, and the molecular basis of NKT17-specific expression of CD138 is unknown (16). As such, it is unclear whether CD138 expression is controlled by RORγt and whether RORγt itself could be a target of CD138 downstream signaling.

To address these questions, we performed a detailed analysis of CD138 expression during T cell development in the thymus. We identified mature CD4 and CD8 double-negative (DN) cells as the only thymocyte population to express CD138. Among CD138^+^ DN cells, *i*NKT cells comprised the vast majority of CD138 expressers (~90%). In agreement with previous findings (15), these CD138^+^ *i*NKT cells were exclusively of the NKT17 lineage. Notably, CD138 expression was associated with but not required for the generation of NKT17 cells because their development remained unimpaired in CD138-deficient (*Sdc1*^–/–^) BALB/c mice and because *Sdc1*^–/–^ NKT17 cells still produced copious amounts of IL-17. Moreover, the ectopic expression of RORγt in NKT1 and NKT2 cells failed to induce CD138 on these cells, thus dissociating CD138 expression from being a potential downstream target of RORγt. Collectively, these findings disentangle NKT17-specific expression of CD138 from NKT17 cell development and propose a model where CD138 expression is a consequence of but not a driving factor for NKT17 cell differentiation.

## Results

### CD138 is expressed on a subset of thymic iNKT cells.

**Figure 1:**
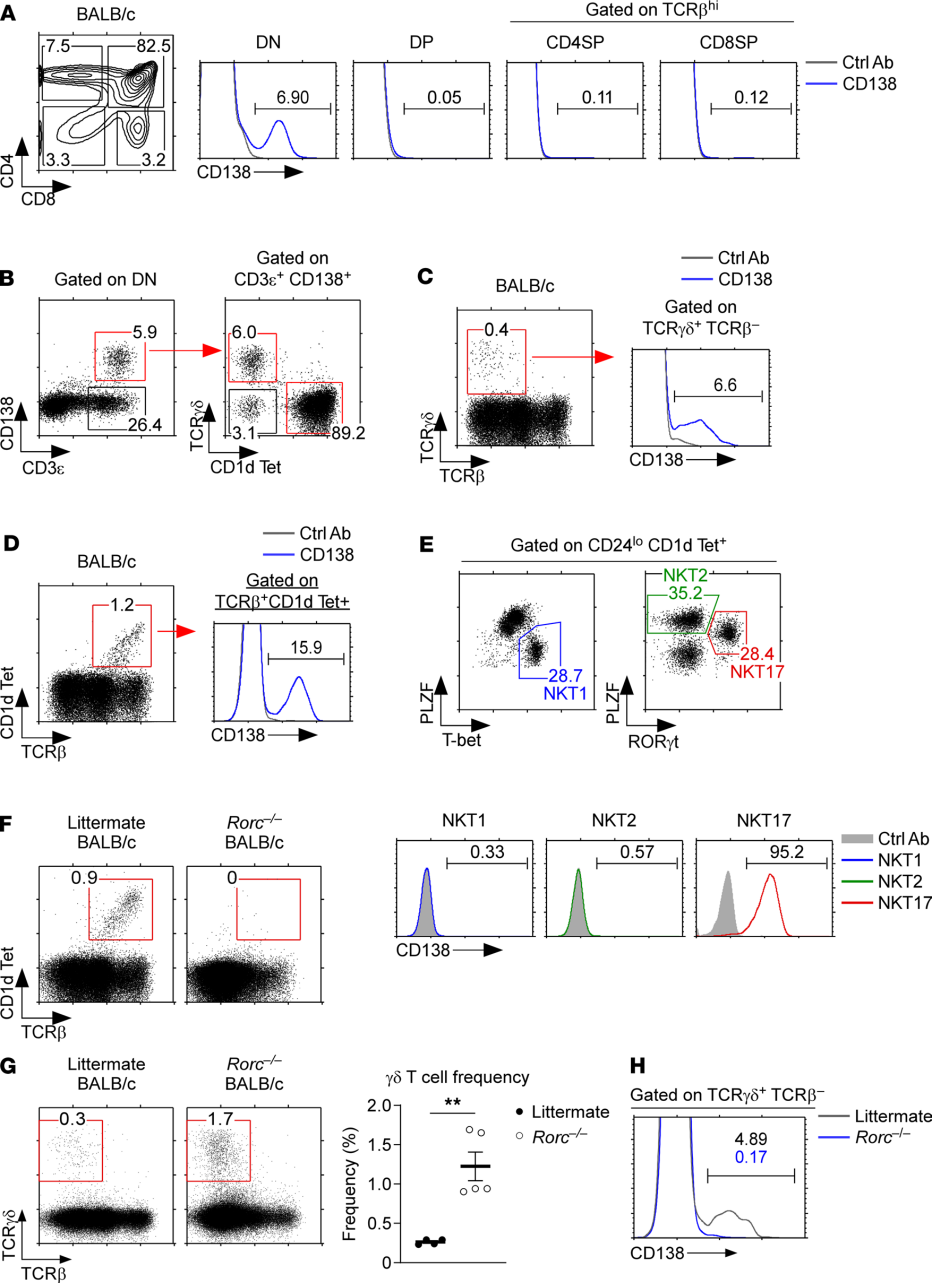
CD138 expression in thymocyte subpopulations. (**A**) CD138 expression was assessed on BALB/c thymocyte subsets, identified by their distinct CD4, CD8, and TCRβ expression. Results are representative of 3 independent experiments. (**B**) CD138 expression among DN thymocytes (left). CD138^+^CD3^+^ DN thymocytes are mostly CD1dTet^+^ *i*NKT cells but also contain conventional αβ and γδ T cells (right). Results represent 3 independent experiments (total 6 BALB/c mice). (**C**) The dot plot and histogram show the identification and CD138 expression of thymic γδ T cells, respectively. Data represent 3 independent experiments (total 8 BALB/c mice). (**D**) The dot plot and histogram show the identification of *i*NKT cells and CD138 expression among thymic *i*NKT cells, respectively. Data summarize 4 independent experiments (total 9 BALB/c mice). (**E**) Individual *i*NKT subsets were identified by intranuclear transcription factor staining (dot plots), which were then assessed for CD138 expression (histograms). Numbers in dot plots indicate frequencies of each *i*NKT subset among CD24^lo^CD1d Tet^+^ thymic *i*NKT cells. The results represent 3 independent experiments (total 4 BALB/c mice). (**F**) Dot plots show *i*NKT cell frequencies in *Rorc*^–/–^ and WT littermate BALB/c thymocytes. Results represent 3 independent experiments with a total of 5 *Rorc*^–/–^ and 4 littermate mice. (**G**) Thymic γδ T cell generation in *Rorc*^–/–^ BALB/c mice. Dot plots are representative, and the bar graph shows the summary of γδ T cell frequencies in *Rorc*^–/–^ and WT littermate BALB/c thymocytes. Results are from 3 independent experiments with a total of 5 *Rorc*^–/–^ and 4 WT littermate mice. (**H**) Histogram shows the frequency of CD138^+^ γδ T cells in *Rorc*^–/–^ and WT littermate BALB/c thymocytes. The results represent 3 independent experiments with a total of 5 *Rorc*^–/–^ and 4 littermate mice. All data are presented as mean ± SEM. *P* values were determined by unpaired Student’s *t* test. **P < 0.01.

To understand the role of CD138 in *i*NKT cell development, we first examined CD138 expression in total thymocytes of BALB/c mice. CD138 was absent on most thymocytes but present on a small subset of DN cells (Figure 1A). DN thymocytes comprise a heterogeneous population of immature and mature T cells (17, 18), and we found that CD138 expression among DN thymocytes was limited to a subpopulation of CD3^hi^ mature T cells (Figure 1B, left). In addition to conventional αβ T cells, mature DN thymocytes also comprise γδ and *i*NKT cells (19, 20). Thus, we gated on CD138^+^CD3^hi^ mature DN cells and asked whether they would correspond to γδ and *i*NKT cells. Most CD138^+^CD3^hi^ DN thymocytes were either γδ or *i*NKT cells (Figure 1B, right). Next, we asked whether all thymic γδ and *i*NKT cells would express CD138. However, this was not the case, as only a small fraction (around 5%) of thymic γδ T cells expressed CD138 (Figure 1C and Supplemental Figure 1A; supplemental material available online with this article; https://doi.org/10.1172/jci.insight.148038DS16). Among *i*NKT cells, we found that approximately 20% of the cells were CD138^+^ (Figure 1D and Supplemental Figure 1B). To correlate CD138 expression with individual *i*NKT subsets, we next examined CD138 expression on NKT1, NKT2, and NKT17 cells, which we identified by their distinct expression of the transcription factors T-bet, PLZF, and RORγt, respectively, as previously described (4, 7). Here, we found CD138 being exclusively expressed on RORγt^+^ NKT17 cells (Figure 1E), which agrees with the seminal report by Hamad and colleagues who identified CD138 as an NKT17 lineage–associated protein (15). Altogether, these results reaffirm CD138 as a surface marker that is uniquely expressed in the NKT17 subset among *i*NKT cells.

To further understand the molecular mechanism that drives CD138 expression on NKT17 cells, we next aimed to assess the role of RORγt in this process. Unfortunately, RORγt is a nonredundant requirement for *i*NKT cell generation in the thymus (21, 22), and RORγt-deficient (*Rorc*^–/–^) BALB/c mice are completely devoid of thymic *i*NKT cells (Figure 1F and Supplemental Figure 1C). Consequently, it was not possible to assess CD138 expression in *i*NKT cells of *Rorc*^–/–^ mice, simply because *i*NKT cells fail to develop in the absence of RORγt. On the other hand, the generation of thymic γδ T cells did not depend on RORγt. In fact, γδ T cell development not only remained intact but resulted in significantly increased frequencies and numbers of γδ T cells in *Rorc*^–/–^ BALB/c mice (Figure 1G and Supplemental Figure 1D). Thus, we could assess the expression of CD138 in thymic γδ T cells, and here we found a conspicuous lack of CD138-expressing γδ T cells when RORγt was absent (Figure 1H and Supplemental Figure 1E). Although these results indirectly support a RORγt requirement for CD138 expression, we consider it unlikely that RORγt expression is sufficient to induce CD138 expression on all T lineage cells. As such, we found that immature double-positive thymocytes, which comprise the main cell population in the thymus, expressed large amounts of RORγt but did not induce CD138 (Figure 1A) (23). These data indicated that CD138 expression is clearly associated with RORγt expression but that cellular factors other than RORγt also play roles in the induction of CD138 expression in T cells.

### iNKT cell development in the absence of CD138.

**Figure 2:**
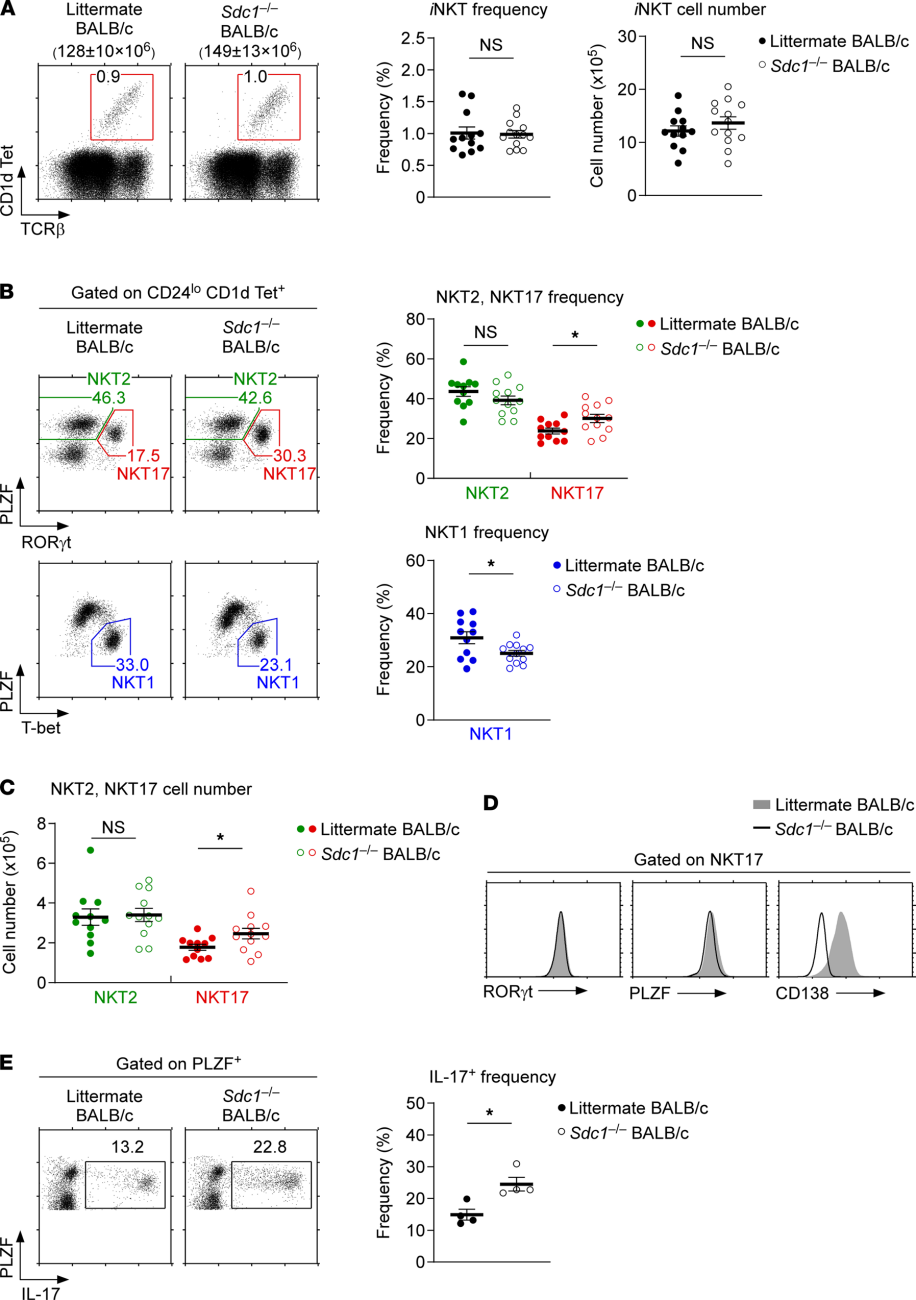
*i*NKT cell development in CD138-deficient mice. (**A**) Identification of thymic *i*NKT cells in *Sdc1*^–/–^ BALB/c mice. The dot plots are representative (left), and the *i*NKT frequency and number graphs show the summary (right) of 8 independent experiments with a total of 13 *Sdc1*^–/–^ and 12 WT littermate BALB/c mice. Total thymocyte numbers are shown on top of the dot plots as mean ± SEM. Numbers in the box show frequencies of *i*NKT cells among total thymocytes. (**B**) *i*NKT subset distribution in *Sdc1*^–/–^ BALB/c thymocytes. The frequencies of NKT1, NKT2, and NKT17 cells were determined by T-bet versus PLZF and RORγt versus PLZF expression. The dot plots are representative, and the graphs show the summary of 7 independent experiments with a total of 12 *Sdc1*^–/–^ and 11 WT littermate BALB/c mice. (**C**) Thymic NKT2 and NKT17 cell numbers were determined in *Sdc1*^–/–^ BALB/c thymocytes. The results show the summary of 7 independent experiments with a total of 12 *Sdc1*^–/–^ and 11 WT littermate BALB/c mice. (**D**) Phenotypic analysis of *Sdc1*^–/–^ NKT17 cells. Thymic NKT17 in *Sdc1*^–/–^ and WT littermate BALB/c mice was assessed for CD138, PLZF, and RORγt expression. Histograms represent 7 independent experiments with a total of 12 *Sdc1*^–/–^ and 11 WT littermate BALB/c mice. (**E**) IL-17 production by PLZF^+^ innate cells in *Sdc1*^–/–^ BALB/c thymocytes. Intracellular IL-17 was assessed among PLZF^+^ cells of freshly isolated *Sdc1*^–/–^ BALB/c thymocytes upon PMA and ionomycin stimulation for 5 hours. Dot plots are representative, and the graph shows the summary of 3 independent experiments with a total of 4 *Sdc1*^–/–^ and 4 WT littermate BALB/c mice. All data are presented as mean ± SEM. *P* values were determined by unpaired 2-tailed Student’s *t* test. *P < 0.05; NS, not significant.

To further examine the role of CD138 in NKT17 cell differentiation, we next assessed thymic *i*NKT cell development in BALB/c mice deficient for the gene *Sdc1*, which encodes CD138 (*Sdc1*^–/–^) (24). Neither the overall T cell development nor the generation of thymic *i*NKT cells were affected by the absence of CD138 (Figure 2A and Supplemental Figure 2). Moreover, the frequencies and numbers of *i*NKT cells in peripheral tissues, such as the liver and spleen, also remained unaffected in *Sdc1*^–/–^ mice. These results indicated that CD138 is not a requirement for the thymic generation and the peripheral maintenance of *i*NKT cells (Supplemental Figure 3A and Supplemental Figure 4A). CD138 deficiency also did not alter the *i*NKT subset composition in the spleen and liver (Supplemental Figure 3B and Supplemental Figure 4B). However, we observed a modest but statistically significant increase in the frequencies and numbers of NKT17 cells in *Sdc1*^–/–^ thymocytes (Figure 2, B and C). Conversely, the frequency of thymic NKT1 cells was significantly decreased in *Sdc1*^–/–^ mice (Figure 2B). Unlike NKT1 and NKT17 cells, however, NKT2 cells remained virtually unaffected in *Sdc1*^–/–^ thymocytes, so that the frequency and number of thymic NKT2 cells did not differ between *Sdc1*^–/–^ and WT littermate mice (Figure 2, B and C). Altogether, CD138 deficiency did not impair but rather promoted the generation of NKT17 cells, albeit at the expense of NKT1 cells.

We next aimed to examine whether CD138-deficient NKT17 cells would differ from CD138-sufficient NKT17 cells regarding their phenotype and function. To this end, we assessed the abundance of the transcription factors PLZF and RORγt in *Sdc1*^–/–^ NKT17 and WT littermate NKT17 cells but did not find any significant difference (Figure 2D). There were no differences in the expression of activation markers and cytokine receptors either (Supplemental Figure 5A). To determine whether NKT17 cells in *Sdc1*^–/–^ BALB/c mice are functionally competent, we next stimulated *Sdc1*^–/–^ BALB/c thymocytes with PMA and ionomycin and assessed their IL-17 production. CD138 deficiency did not impair IL-17 production, and consistent with an increase in NKT17 cell frequencies, we found that IL-17 expression was increased in PLZF^+^ thymocytes of *Sdc1*^–/–^ BALB/c mice (Figure 2E). Collectively, these results showed that CD138 was not required for the generation or effector function of NKT17 cells.

### Phenotypic and functional analyses of thymic Sdc1^–/–^ NKT17 cells.

**Figure 3:**
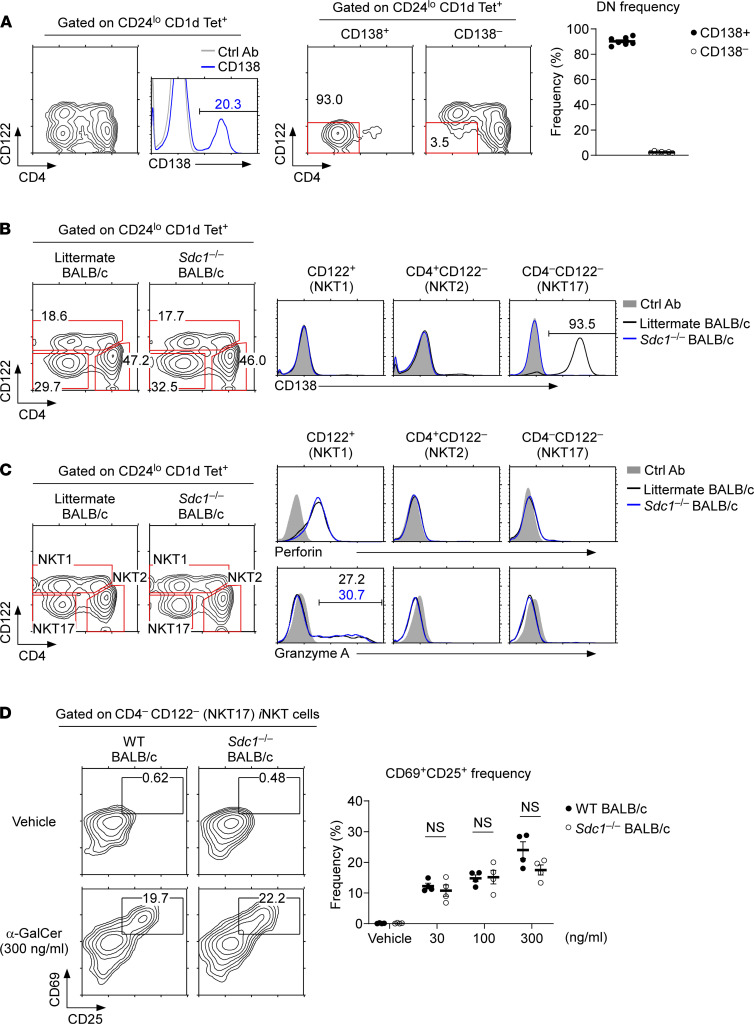
Functional and phenotypical characterization of *Sdc1*^–/–^ *i*NKT cells. (**A**) Identification of thymic NKT17 cells based on CD122 and CD4 expression. CD138^+^ and CD138^–^ *i*NKT cells were assessed for surface CD122 and CD4 expression. Data are representative of 5 independent experiments with a total of 7 BALB/c mice. (**B**) *i*NKT subset classification based on CD122 and CD4 expression. Thymic *i*NKT cells of *Sdc1*^–/–^ and WT littermate BALB/c thymocytes were assessed for CD122 and CD4 expression, visualizing the 3 subsets of NKT1 (CD122^+^), NKT2 (CD122^–^CD4^+^), and NKT17 (CD122^–^ CD4^–^) cells (left). CD138 expression was assessed in the indicated *i*NKT subsets of *Sdc1*^–/–^ and WT littermate BALB/c thymocytes. Data are representative of 2 independent experiments. (**C**) *i*NKT subsets were identified in fixed and permeabilized thymocytes of *Sdc1*^–/–^ and WT littermate BALB/c mice based on CD122 and CD4 expression (left). Intracellular perforin and granzyme A expression were then assessed in each of the indicated *i*NKT subsets. Contour plots and histograms are representative of 3 independent experiments. (**D**) Surface CD69 and CD25 expression was assessed on NKT17 cells upon overnight in vitro stimulation of *Sdc1*^–/–^ and WT littermate BALB/c thymocytes with the indicated amounts of α-GalCer. Contour plots are representative and bar graph shows the summary of 3 independent experiments. All data are presented as mean ± SEM. *P* values were determined by unpaired 2-tailed Student’s *t* test. NS, not significant.

To further examine the role of CD138 in NKT17 cells, we wished to identify NKT17 cells by surface markers other than CD138. *Sdc1*^–/–^ NKT17 cells lack CD138, so CD138 cannot be employed as a marker for NKT17 cells. To this end, we stained thymic *i*NKT cells for CD4 and CD122, a combination of 2 surface molecules that was previously reported to discriminate the 3 *i*NKT subsets (25). Indeed, we found that NKT17 cells, as identified by CD138 expression, were highly enriched in the CD4^–^CD122^–^ DN population (Figure 3A). NKT1 and NKT2 cells, on the other hand, are CD138 negative, and these subsets were found in the CD4^+^ and CD122^+^ populations, but they were conspicuously absent among DN cells (Figure 3A). To test whether the DN subset would indeed correspond to NKT17 cells, we next assessed surface CD138 expression on individual *i*NKT subsets. CD122^+^ cells corresponded to NKT1 cells, whereas CD4^+^CD122^–^ cells corresponded to NKT2 cells, and neither of these subsets expressed CD138 (Figure 3B). DN cells, however, were highly enriched in CD138^+^ *i*NKT cells, indicating that they can be considered as NKT17 cells. In agreement, we found that RORγt was highly expressed in DN but not in CD4^+^ or CD122^+^ *i*NKT cells (Supplemental Figure 5B). Altogether, the visualization of *i*NKT subsets by CD4 and CD122 permitted the identification of NKT17 cells independent of CD138 expression.

Based on CD4 and CD122 staining, we next assessed intracellular perforin and granzyme A expression in thymic *i*NKT subsets of *Sdc1*^–/–^ and WT littermate mice. Both perforin and granzyme A expression are linked with the cytotoxic function of *i*NKT cells (26), and we found them to be exclusively expressed in NKT1 cells (Figure 3C). NKT17 cells of *Sdc1*^–/–^ and WT mice did not express these cytolytic molecules (Figure 3C). These results indicated that the lack of CD138 in *Sdc1*^–/–^ NKT17 cells did not result in their acquisition of NKT1-like effector functions, and these data reaffirmed that NKT17 cells retained their subset-specific characteristics independently of CD138.

Lastly, we asked whether CD138 plays a role in NKT17 activation such that the lack of CD138 would alter the antigen responsiveness of NKT17 cells. To address this question, we stimulated thymocytes of *Sdc1*^–/–^ and WT mice with increasing amounts of α-GalCer and assessed the induction of CD69 and CD25, 2 prominent activation markers (27), on *i*NKT cells after overnight culture in vitro. α-GalCer stimulation induced a marked increase in CD25 and CD69 expression on *i*NKT cells of *Sdc1*^–/–^ and WT mice (Supplemental Figure 5C). However, we failed to find any significant difference between *Sdc1*^–/–^ and WT NKT17 cells (Figure 3D). Thus, the lack of CD138 did not affect the activation threshold or the antigen responsiveness of NKT17 cells. Collectively, we found that *Sdc1*^–/–^ NKT17 cells did not significantly differ in their phenotype and effector molecule expression compared with CD138-expressing WT NKT17 cells.

### CD138 deficiency does not affect innate CD8 T cell generation in the thymus.

**Figure 4:**
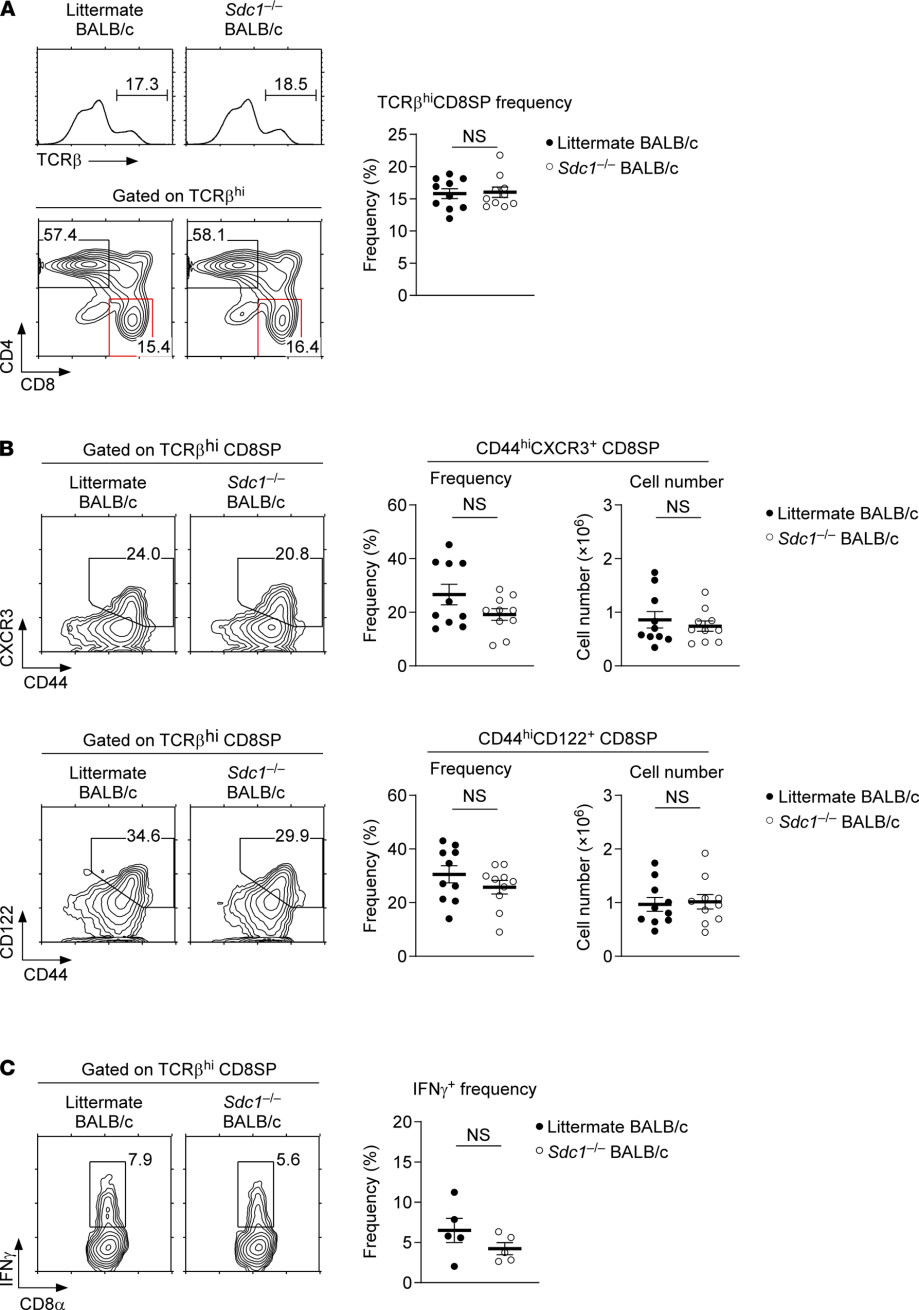
Thymocyte development in CD138-deficient mice. (**A**) T cell development in the thymus of *Sdc1*^–/–^ BALB/c mice. Mature thymocytes were identified by high levels of TCRβ expression and then further assessed for CD4 and CD8 coreceptor expression. Histograms and contour plots (left) are representative, and the graph showing the frequency of CD8 T cells (right) is a summary of 6 independent experiments with a total of 10 *Sdc1*^–/–^ and 10 WT littermate BALB/c mice. (**B**) Innate-type marker expression and cell numbers of CD8SP thymocytes of *Sdc1*^–/–^ BALB/c mice. CD44 versus CXCR3 (top) and CD44 versus CD122 (bottom) expression profiles, and the frequencies and numbers of innate-type cells were assessed in TCRβ^hi^ CD8SP thymocytes of *Sdc1*^–/–^ and WT littermate BALB/c mice. The contour plots represent and the graphs summarize 6 independent experiments with 10 *Sdc1*^–/–^ and 10 WT littermate BALB/c mice. (**C**) IFN-γ production by CD8SP cells of *Sdc1*^–/–^ BALB/c thymocytes. IFN-γ was assessed among TCRβ^hi^CD8SP freshly isolated *Sdc1*^–/–^ BALB/c thymocytes upon PMA and ionomycin stimulation for 5 hours. Data are representative of 3 independent experiments with a total of 5 *Sdc1*^–/–^ and 5 WT littermate BALB/c mice. All data are presented as mean ± SEM. *P* values were determined by unpaired 2-tailed Student’s *t* test. NS, not significant.

BALB/c mice contain a large fraction of NKT2 cells that serve as a major source of intrathymic IL-4 (4, 7, 28), which drives the generation of innate CD8 T cells in the thymus (4). Thus, BALB/c mice produce large numbers of innate-phenotype CD8 T cells (28, 29), and innate CD8 T cells produce copious amounts of IFN-γ to create a proinflammatory Th1 environment (30). Whether CD138 is involved in innate CD8 T cell generation is not known. However, we considered it important to assess this possibility because CD138 alters the thymic *i*NKT subset composition. To this end, we next analyzed thymocyte development in *Sdc1*^–/–^ and WT littermate BALB/c mice. The CD4 versus CD8 thymocyte profile and the frequency of TCRβ^hi^ CD8 single-positive (CD8SP) thymocytes remained unaltered in *Sdc1*^–/–^ mice, suggesting that the generation of conventional and innate CD8 T cells was comparable to that of CD138-sufficient WT littermate mice (Figure 4A). Indeed, the frequency and number of CD44^hi^CD122^+^ and CD44^hi^CXCR3^+^ CD8SP cells that mostly corresponded to innate CD8 T cells did not significantly differ between *Sdc1*^–/–^ and WT littermate BALB/c mice (Figure 4B). To further confirm that CD138 deficiency did not impair the effector function of *Sdc1*^–/–^ innate CD8 T cells, we also stimulated *Sdc1*^–/–^ and WT littermate BALB/c thymocytes with PMA and ionomycin and assessed IFN-γ production in CD8SP thymocytes. As expected, CD8SP thymocytes from *Sdc1*^–/–^ BALB/c mice produced similar amounts of IFN-γ as WT littermate BALB/c CD8SP cells (Figure 4C), further demonstrating that CD138 is not required for the development or effector function of innate CD8 T cells.

**Figure 5:**
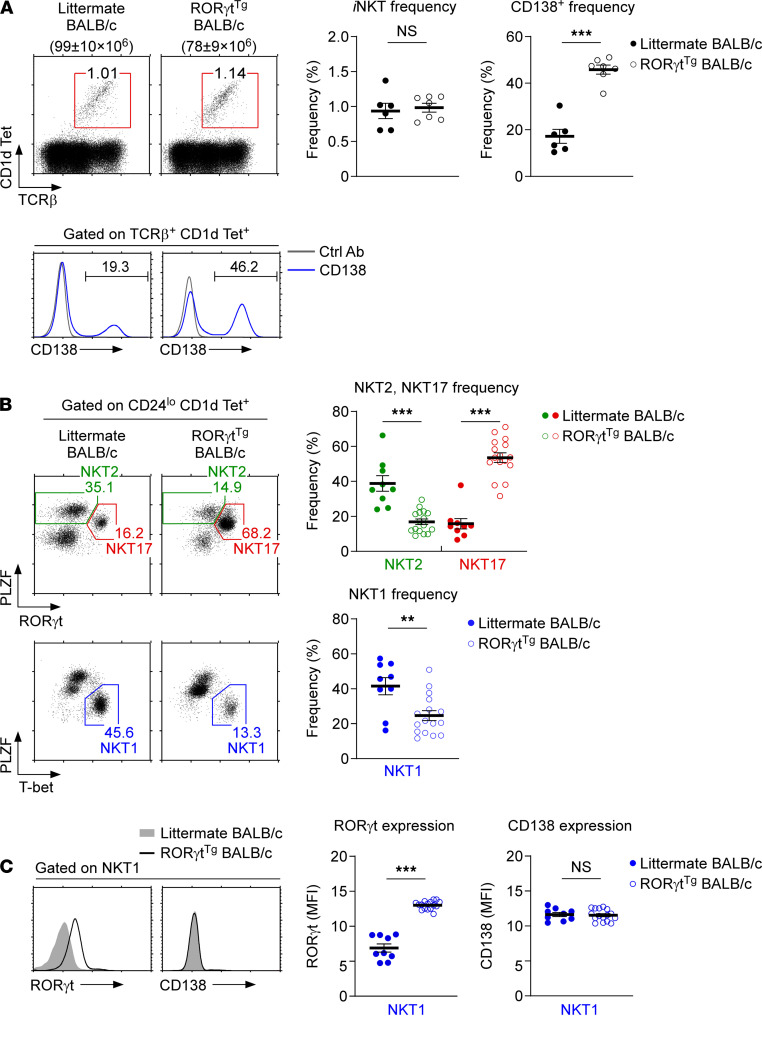
*i*NKT subset differentiation in RORγt^Tg^ BALB/c mice. (**A**) CD138 expression on thymic *i*NKT cells of RORγt^Tg^ and WT littermate BALB/c mice. The dot plots identify and show the frequency of thymic *i*NKT cells (top), and the histograms show CD138 expression among *i*NKT cells (bottom). Graphs (right) show the frequency of *i*NKT cells among total thymocytes and the frequency of CD138^+^ cells among thymic *i*NKT cells. Data show summary of 2 independent experiments with a total of 7 RORγt^Tg^ and 6 WT littermate BALB/c mice. (**B**) Thymic *i*NKT subset composition of RORγt^Tg^ and WT littermate BALB/c mice. The dot plots show the frequencies of each *i*NKT subset identified by PLZF versus T-bet and PLZF versus RORγt staining (left). The graphs show the frequencies of NKT1, NKT2, and NKT17 cells among thymic mature *i*NKT cells (right). Data summarize 4 independent experiments with a total of 16 RORγt^Tg^ and 9 WT littermate BALB/c mice. (**C**) RORγt and CD138 expression in T-bet^+^ NKT1 cells of RORγt^Tg^ and WT littermate BALB/c mice. The histograms show RORγt and CD138 expression in thymic NKT1 cells (left). The graphs show the MFI of RORγt and CD138 expression in thymic NKT1 cells of the indicated mice (right). Data are the summary of 4 independent experiments with a total of 16 RORγt^Tg^ and 9 WT littermate BALB/c mice. All data are presented as mean ± SEM. *P* values were determined by unpaired 2-tailed Student’s *t* test. **P < 0.01; ***P < 0.001; NS, not significant.

*Forced expression of RORγt alters the subset composition of thymic iNKT cells*. RORγt is the transcription factor that specifies NKT17 lineage differentiation in *i*NKT cells (22). Because CD138 is exclusively expressed on RORγt^+^ NKT17 cells (15), we next asked whether forced expression of RORγt was sufficient to induce CD138 expression on *i*NKT cells. To this end, we examined thymic *i*NKT cells of RORγt-transgenic (RORγt^Tg^) and WT littermate BALB/c mice for surface CD138 expression. RORγt^Tg^ mice have been previously described (23), and they express the murine RORγt cDNA under the control of the proximal *Lck* promoter. Accordingly, all thymocytes, including thymic *i*NKT cells, are forced to express RORγt (Supplemental Figure 6A). Although RORγt overexpression did not significantly alter the frequency of thymic *i*NKT cells (Figure 5A), strikingly, the frequency of CD138^+^ cells was dramatically increased among *i*NKT cells (Figure 5A). In agreement with the effect on *i*NKT cells, the forced expression of RORγt also dramatically increased the frequency of CD138^+^ cells among thymic γδ T cells (Supplemental Figure 6B). Thus, the abundance of RORγt correlated with the frequency of CD138^+^ T cells and presumably drove their generation in the thymus.

To determine whether the increase in CD138^+^ *i*NKT cell frequency is associated with an increase in NKT17 cells, we next assessed the *i*NKT subset composition in RORγt^Tg^ and WT littermate BALB/c thymocytes (Figure 5B and Supplemental Figure 6C). Intracellular staining for RORγt and PLZF showed that the frequency of NKT17 cells, which correspond to PLZF^int^RORγt^hi^ *i*NKT cells, was indeed dramatically increased in RORγt^Tg^ thymocytes (Figure 5B). Notably, the increase in NKT17 cell frequency and cell number was concomitant with the loss of PLZF^hi^ NKT2 cells and T-bet^+^ NKT1 cells (Figure 5B and Supplemental Figure 6C). These results document that forced expression of RORγt altered the thymic *i*NKT subset composition and further indicate that RORγt expression was sufficient to impose NKT17 lineage fate on developing *i*NKT cells.

We next aimed to assess whether CD138 expression is a direct target of RORγt. CD138 expression could have been upregulated by increased RORγt activity, but also as a consequence of NKT17 lineage differentiation, independently of RORγt. In this regard, we wished to examine whether the forced expression of RORγt would be sufficient to induce the ectopic expression of CD138 in NKT1 cells because NKT1 cells express neither RORγt nor CD138 (15). We confirmed that NKT1 cells of RORγt^Tg^ mice coexpressed RORγt with T-bet (Figure 5C). However, such RORγt^+^ NKT1 cells still remained negative for CD138 (Figure 5C). This was also the case for NKT2 cells in RORγt^Tg^ mice, where RORγt expression was significantly increased but CD138 expression was not induced (Supplemental Figure 7). Altogether, these results suggest that RORγt expression alone, at least in the amounts found in RORγt^Tg^ NKT1 and RORγt^Tg^ NKT2 cells, is not sufficient to induce CD138 expression. Whether further increasing the abundance of transgenic RORγt would possibly induce CD138 expression in NKT1 and NKT2 cells remains to be tested. Collectively, CD138 is induced upon NKT17 lineage commitment and its expression is associated with but not required for NKT17 cell differentiation.

**Figure 6:**
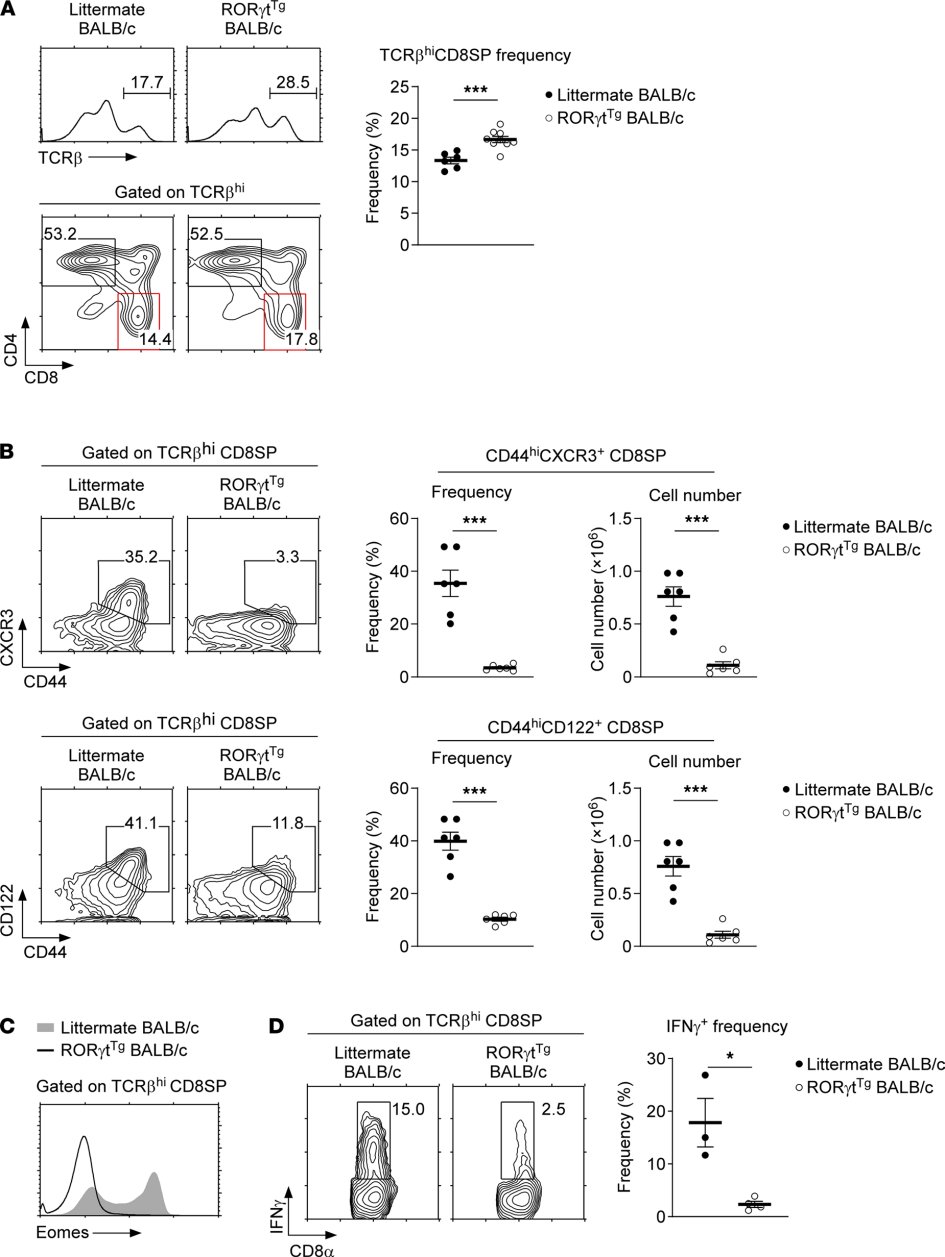
Lack of innate CD8 T cells in RORγt^Tg^ BALB/c thymocytes. (**A**) T cell development in the thymus of RORγt^Tg^ BALB/c mice. Mature thymocytes were identified by high levels of TCRβ expression and then further assessed for CD4 and CD8 coreceptor expression. Histograms and contour plots (left) are representative, and the graph showing the frequency of CD8SP T cells (right) is a summary of 3 independent experiments with a total of 9 RORγt^Tg^ and 6 WT littermate BALB/c mice. (**B**) Innate-type marker expression and cell numbers of CD8SP thymocytes of RORγt^Tg^ BALB/c mice. CD44 versus CXCR3 and CD44 versus CD122 expression profiles and the frequencies and numbers of innate-type cells were assessed in TCRβ^hi^ CD8SP thymocytes of RORγt^Tg^ and WT littermate BALB/c mice. The contour plots represent and the graphs summarize 2 independent experiments with a total of 6 RORγt^Tg^ and 6 WT littermate BALB/c mice. (**C**) Intranuclear staining for Eomes in mature CD8SP thymocytes of RORγt^Tg^ and WT littermate BALB/c mice. The histogram is representative of 2 independent experiments with a total of 4 RORγt^Tg^ and 3 WT littermate BALB/c mice. (**D**) IFN-γ production by mature CD8SP cells of RORγt^Tg^ and WT littermate BALB/c thymocytes that were stimulated with PMA and ionomycin for 5 hours in the presence of brefeldin A. Data summarize 2 independent experiments with a total of 4 RORγt^Tg^ and 3 WT littermate BALB/c mice. All data are presented as mean ± SEM. *P* values were determined by 2-tailed unpaired Student’s *t* test. *P < 0.05; ***P < 0.001.

*Loss of innate CD8 T cells in RORγt^Tg^ thymocytes*. Because the forced expression of RORγt potently suppressed the generation of NKT2 cells, we next aimed to assess whether the development of innate CD8 T cells, which depend on NKT2 cells, would also be impaired in RORγt^Tg^ mice (4, 29, 31). To this end, we first analyzed the CD4 versus CD8 profile and the frequency of TCRβ^+^ CD8SP thymocytes in RORγt^Tg^ and WT littermate mice. Although the generation of mature CD8 thymocytes was not impaired in RORγt^Tg^ BALB/c mice (Figure 6A), the frequency and number of CD44^hi^CD122^+^ and CD44^hi^CXCR3^+^ innate phenotype CD8SP cells were dramatically reduced (Figure 6B). Innate CD8 T cells are also marked by high expression of the transcription factor Eomes, and we found that the frequency of Eomes^+^ cells was substantially reduced among CD8SP thymocytes of RORγt^Tg^ mice (Figure 6C). Thus, consistent with the requirement for NKT2 cells, which were substantially diminished in RORγt^Tg^ thymocytes, innate CD8 T cells failed to develop in RORγt^Tg^ mice.

To further confirm that RORγt^Tg^ CD8 T cells indeed lack innate T cell function, we next stimulated RORγt^Tg^ and WT littermate BALB/c thymocytes with PMA and ionomycin to assess IFN-γ production in CD8SP thymocytes. As expected, CD8SP thymocytes from RORγt^Tg^ mice showed substantially reduced amounts of IFN-γ production compared with WT littermate BALB/c CD8SP cells (Figure 6D). These results document the biological impact of forced RORγt expression on innate CD8 T cells, potentially by altering the subset composition of thymic *i*NKT cells. At this point, we also cannot exclude a direct effect of forced RORγt expression on innate CD8 T cell differentiation in a cell-intrinsic manner, and we aim to address this issue in our future studies.

**Figure 7:**
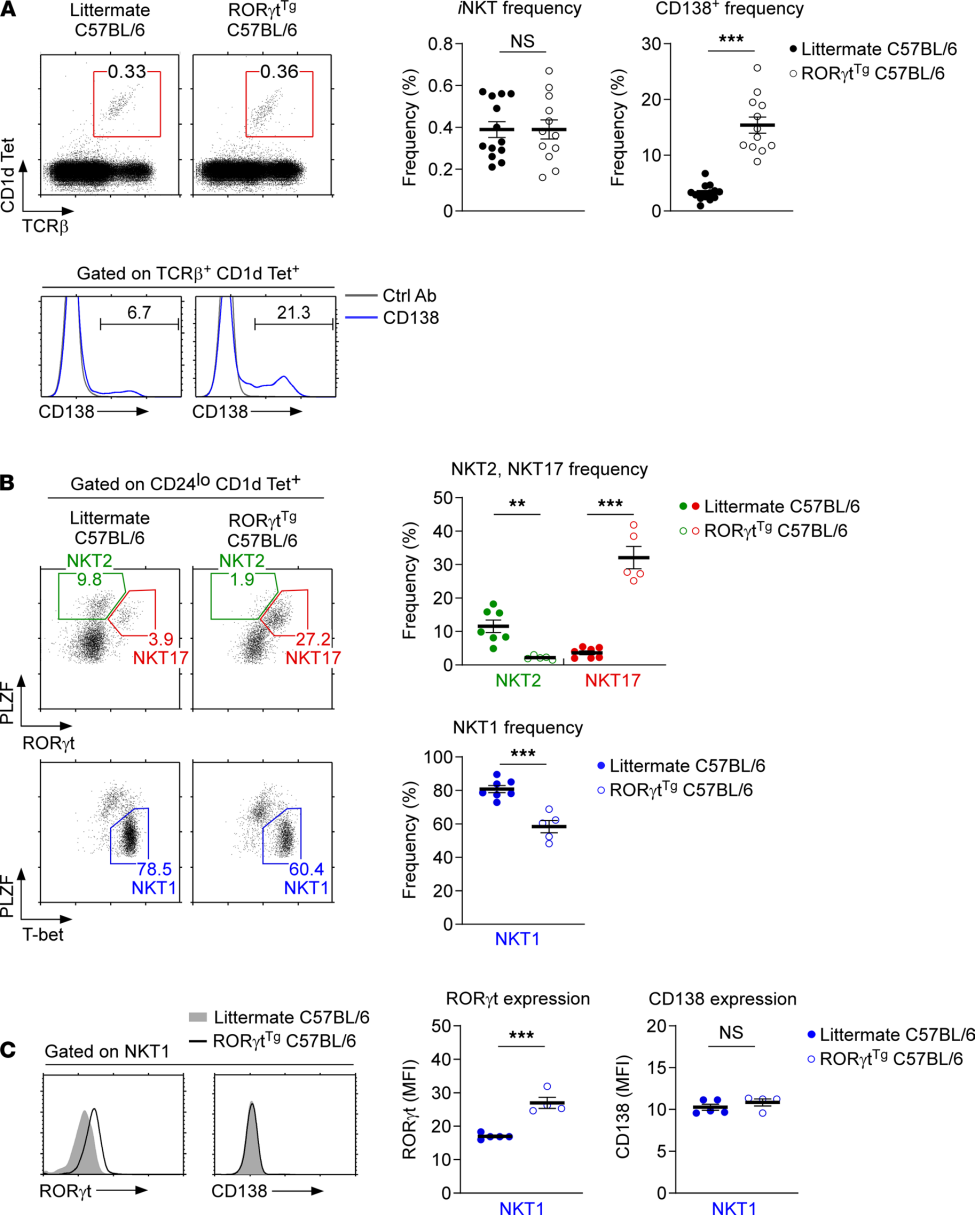
*i*NKT subset differentiation in RORγt^Tg^ C57BL/6 mice. (**A**) CD138 expression on thymic *i*NKT cells of RORγt^Tg^ and WT littermate C57BL/6 mice. The dot plots identify and show the frequency of thymic *i*NKT cells (top), and the histograms show CD138 expression among *i*NKT cells of C57BL/6 mice (bottom). The graphs show the frequency of *i*NKT cells among total thymocytes (left) and the frequency of CD138^+^ cells among thymic *i*NKT cells (right). Data summarize 6 independent experiments with a total of 12 RORγt^Tg^ and 13 WT littermate C57BL/6 mice. (**B**) Thymic *i*NKT subset composition of RORγt^Tg^ and WT littermate C57BL/6 mice. The dot plots show the frequencies of each *i*NKT subset identified by PLZF versus RORγt and PLZF versus T-bet staining (left). The graphs show the frequencies of NKT1, NKT2, and NKT17 cells among thymic mature *i*NKT cells (right). Data summarize 3 independent experiments with a total of 5 RORγt^Tg^ and 7 WT littermate C57BL/6 mice. (**C**) RORγt and CD138 expression in T-bet^+^ NKT1 cells of RORγt^Tg^ and WT littermate C57BL/6 mice. Histograms show RORγt and CD138 expression in thymic NKT1 cells (left). The graphs show the MFI of RORγt and CD138 expression in thymic NKT1 cells of the indicated mice (right). Data summarize 2 independent experiments with a total of 4 RORγt^Tg^ and 5 WT littermate C57BL/6 mice. All data are presented as mean ± SEM. *P* values were determined by unpaired 2-tailed Student’s *t* test. **P < 0.01; ***P < 0.001; NS, not significant.

*Forced expression of RORγt promotes NKT17 cell differentiation in C57BL/6 mice*. Forced expression of RORγt dramatically altered the *i*NKT subset composition in BALB/c mice. Thus, we wished to determine whether this was also the case in C57BL/6 mice. The development and differentiation of *i*NKT cells vary significantly depending on the genetic background (4, 28), and we considered it important to confirm the effect of RORγt overexpression in C57BL/6 mice. In agreement with the results from RORγt^Tg^ BALB/c mice, RORγt^Tg^ C57BL/6 mice did not display significant alterations in the overall frequency of thymic *i*NKT cells (Figure 7A). The frequency of CD138^+^ *i*NKT cells, however, was dramatically increased, corroborating the notion that forced expression of RORγt imposes NKT17 lineage fate on developing *i*NKT cells (Figure 7A). Indeed, assessing the subset composition of RORγt^Tg^ and WT littermate *i*NKT cells revealed a dramatic increase in the frequency and number of NKT17 cells, concomitant with a substantial decrease in NKT1 cells (Figure 7B and Supplemental Figure 8). Because RORγt overexpression did not alter the overall frequency of *i*NKT cells, these results indicate that forced RORγt expression specifically affected *i*NKT subset fate but not the number of *i*NKT cells, and that RORγt was sufficient to redirect *i*NKT development into NKT17 lineage cells. Moreover, few of the remaining NKT1 cells that coexpress RORγt and T-bet failed to induce CD138 expression (Figure 7C), indicating that CD138 was stringently associated with NKT17 cell differentiation but not necessarily downstream of RORγt. Collectively, these results confirmed RORγt as a subset-specifying factor in BALB/c and C57BL/6 mice and identified CD138 as a surface marker whose expression is associated with but not induced upon RORγt expression.

## Discussion

The molecular mechanism that drives the trifurcation of developing *i*NKT cells into distinct *i*NKT effector subsets remains incompletely understood. Because CD138 is absent on immature *i*NKT cells but exclusively expressed on mature NKT17 cells (15), here we examined the requirement for CD138 in NKT17 cell generation. We also asked whether CD138 expression is a target of RORγt, a transcription factor that specifies NKT17 lineage fate (10, 16). Our results confirmed CD138 as a highly selective marker associated with NKT17 cells (15), but we also report that CD138 was not required for the phenotypic or functional maturation of IL-17–producing *i*NKT cells. Moreover, the forced expression of RORγt was sufficient to impose NKT17 subset fate on thymic *i*NKT cells but without a significant increase in total *i*NKT cell numbers. These results document RORγt as a positive regulator of NKT17 cell generation that promotes NKT17 cell differentiation during thymic *i*NKT cell differentiation. Curiously, not all *i*NKT cells in such RORγt^Tg^ thymocytes had upregulated CD138 expression, indicating that factors other than RORγt also contribute to the NKT17-specific expression of CD138. Thus, CD138 might accompany NKT17 cell differentiation and might be associated with RORγt expression, but CD138 expression itself is presumably not a direct molecular target of RORγt.

Among the various *i*NKT subsets, NKT17 cells have attracted much interest because they are considered the major intrathymic αβ T cells that produce IL-17 (16). IFN-γ is the signature cytokine of NKT1 cells, but IFN-γ is also expressed by innate CD8 T cells (30). IL-4 is highly expressed by NKT2 cells, but it can also be produced by CD44^hi^ memory-phenotype CD4SP cells (32, 33). Intrathymic IL-17, however, is primarily produced by NKT17 cells, thus establishing a unique role for NKT17 cells among thymic αβ T cells. Along these lines, the developmental program of NKT17 cells also contains some unique features (16). For example, the runt family transcription factor Runx1 plays a nonredundant role specifically in NKT17 cell generation (34), and the transcriptional repressor NKAP exclusively promotes NKT17 cell generation, as illustrated in the dramatically diminished numbers of NKT17 cells in NKAP-deficient mice (35). As previously reported and reaffirmed in our study, NKT17 cells are the only expressers of CD138 among thymic *i*NKT cells (15). Despite its selective expression on NKT17 cells, however, CD138 was not required to specify NKT17 subset fate or to produce IL-17. Thus, the biological significance of CD138 expression on NKT17 cells remains unclear.

CD138 is a cell-surface heparan sulfate proteoglycan that is primarily expressed on epithelial cells but is also found on immune cells (36). Among others, CD138 is expressed on end-differentiated plasma cells and on a subset of IL-17–producing γδ T (Tγδ 17) cells (37, 38). CD138 is also highly expressed on myeloma cells such that it is not only used as a biomarker but also considered as a target for treatment of multiple myeloma (39). Functionally, CD138 expression has been proposed to promote the survival and homeostasis of mature plasma cells as well as peripheral Tγδ 17 cells (37, 38). Notably, such a prosurvival effect was associated with increased proliferation in Tγδ-17 cells but in a manner that is T cell intrinsic and independent of CD138 expression on nonhematopoietic cells (37). Indeed, CD138 binds antiapoptotic factors, such as APRIL (40), and it can interact with cytokines, chemokines, and growth factors, which promote the survival of CD138-expressing cells (41). While a cell-intrinsic effect of CD138 is evidently a major pathway to promote the survival of CD138^+^ immune cells, CD138 also exerts its effects in a cell-extrinsic fashion. As such, the extracellular domain of CD138 can be cleaved from the cell surface and shed into the environment, a process that is controlled by the small GTPase Rab5 and mediated by surface secretases (36, 42). Because soluble CD138 retains its biologically active heparan sulfate chains, secreted CD138 proteins can bind to and modulate the activity of soluble factors, including cytokines and growth factors. Along these lines, the increased abundance of soluble CD138 has often been associated with inflammation and leukocyte migration, which could either mitigate or exacerbate immune responses (41, 43). However, interpreting the biological implication of increased soluble CD138 expression is not always straightforward because CD138 interacts with multiple molecules in a context-dependent manner.

It is not clear whether, and if so how, CD138 would affect the biology of NKT17 cells because the generation of NKT17 cells is not adversely affected in mice that are deficient in CD138. Moreover, it also remains unclear to us why the lack of CD138, which is specifically expressed on NKT17 cells, would promote, albeit modestly, the differentiation of NKT17 cells. Because the NKT1 cell frequency is decreased in the absence of CD138, it is tempting to postulate that NKT1 and NKT17 cells, but not NKT2 cells, branch out from a common precursor by alternative lineage choice. Indeed, such a model was recently suggested based on single-cell RNA-Seq analysis (44). Accordingly, NKT2 cells represent a developmental branching point for NKT1 and NKT17 cells, and CD138 could potentially influence subset differentiation at this point by suppressing NKT17 but promoting NKT1 cell generation. Consequently, CD138 expression could be a homeostatic tool to self-limit the expansion of NKT17 cells by promoting NKT1 lineage choice and act as a negative regulatory feedback signal that controls the size of the NKT17 cell pool in the thymus. How such a CD138-mediated mechanism can be understood in the context of the current models of *i*NKT subset differentiation will need further study.

Currently, 2 distinct but not mutually exclusive models are proposed to explain thymic *i*NKT cell differentiation. The linear differentiation model posits that *i*NKT cell effector functions are acquired along a well-explored pathway of thymic differentiation that is defined by CD44 and NK1.1 expression (45). The lineage-diversification model (4), on the other hand, proposes that a common *i*NKT precursor gives rise to 3 distinct *i*NKT subsets. According to the lineage diversification model, there is no precursor-progeny relationship between the subsets, and their specific effector functions depend on the *i*NKT subset identity. Regardless of the model, however, it is important to know what cellular signals drive the acquisition of specific effector functions at a specific developmental stage or for a particular subset of *i*NKT cells. Much progress has been made in this area with the help of genetic mouse models (4). Specifically, the preferential loss or increase of a particular *i*NKT subset in different mouse strains has helped to assess the molecular machinery that drives *i*NKT lineage fate (46). C57BL/6 mice, for example, contain mostly NKT1 cells, whereas BALB/c mice produce greater frequencies of NKT2 and NKT17 cells (4, 46). The transcription factor KLF13 was found to increase NKT2 cell differentiation in BALB/c mice (28), but it remains unclear why BALB/c mice would express larger amounts of KLF13 and what signals in BALB/c mice would increase KLF13 expression. The increased frequency of NKT17 cells in *Sdc1*^–/–^ BALB/c mice now suggests that CD138 is another factor that influences *i*NKT subset-specific differentiation. However, a comprehensive model that integrates all these different factors for *i*NKT subset specification is currently not available. Nonetheless, it is evident that the expression of signature transcription factors is the main driver of *i*NKT subset differentiation, and the forced expression of RORγt, as shown in this study, is sufficient to promote NKT17 fate onto developing thymic *i*NKT cells.

Altogether, the current study untangles the expression of CD138 from the differentiation of NKT17 cells and demonstrates that NKT17 subset generation is driven by mechanisms independent of CD138. Thus, CD138 is certainly a marker of RORγt expression in *i*NKT cells but it is not a requirement for RORγt induction or IL-17 expression. Notably, immature double-positive thymocytes lack CD138 expression but they express large amounts of RORγt (23, 47). These results further indicate that RORγt expression itself is not sufficient to drive CD138 and/or IL-17 expression. In fact, the cellular and developmental context of RORγt expression is critical to impose effector function and drive subset specification during T cell development. Identification of these factors is the aim of our future studies.

## Methods

### Mice.

BALB/c and C57BL/6 (B6) mice of both sexes were obtained from Charles River Laboratories and analyzed between 6 and 12 weeks of age. CD138-deficient mice (*Sdc1*^–/–^) were previously described (24) and maintained on a BALB/cAnNCrl background (Charles River Laboratories) at the NIH. RORγt^Tg^ mice were generated in-house (23) and either maintained on a C57BL/6 background or backcrossed to BALB/cAnNCrl mice. RORγt-deficient mice (*Rorc*^–/–^) were obtained from The Jackson Laboratory (47) and backcrossed to BALB/cAnNCrl mice.

### Flow cytometry.

Single-cell suspensions were prepared from the thymus of the experimental mice and stained with fluorescence-conjugated antibodies as previously described (48). After staining, cells were analyzed using LSRFortessa, LSRFortessa X-20, or LSR II flow cytometers (BD Biosciences) and software designed in-house at the Experimental Immunology Branch, National Cancer Institute. Live cells were identified using forward-scatter exclusion of dead cells stained with propidium iodide. For intracellular staining, cells were first stained with Ghost Dye Violet 510 (Tonbo Biosciences) for dead-cell exclusions, followed by surface staining and fixing with intracellular fixation buffer (eBioscience) or Foxp3 fixation buffer (eBioscience). After fixation, cells were permeabilized using the Foxp3 intracellular staining kit according to the manufacturer’s instructions (Thermo Fisher eBioscience). The following antibodies were used for staining: TCRβ (eBioscience, clone H57-597), CD4 (Tonbo Biosciences, clone GK1.5), CD8 (Tonbo Biosciences, clone 53-67), CD24 (Biolegend, clone M1/69), CD138 (BD Bioscience, clone 281-2), TCRγδ (Biolegend, clone GL3), CD122 (eBioscience, clone TM-β1), CXCR3 (eBioscience, clone CXCR3-173), CD44 (Tonbo Biosciences, clone IM7), CD69 (Biolegend, clone H1.2F3), CD5 (eBioscience, clone 53-7.3), IL-7Rα (eBioscience, clone A7R34), CD132 (BD Bioscience, clone 4G3), CD25 (eBioscience, clone PC61.5), IL-17 (eBioscience, clone eBio17B7), IFN-γ (Biolegend, clone XMG1.2), PLZF (Biolegend, clone 9E12), RORγt (BD Bioscience, clone Q31-378), T-bet (eBioscience, clone eBio4B10), Eomes (eBioscience, clone Dan11mag), perforin (Biolegend, clone A16009A), and granzyme A (eBioscience, clone GzA-3G8.5). CD1d tetramers loaded with PBS-57 and unloaded controls were obtained from the NIH tetramer facility (Emory University, Atlanta, GA).

### Lymphocyte isolation.

Lymphocytes were processed into single-cell suspensions from the thymus, spleen, and liver as previously described (7). For enrichment of liver lymphocytes, livers of the indicated mice were gently pressed through 70 μm cell strainers (BD Biosciences), and the tissue suspensions were washed 2 times in ice-cold PBS. Cell pellets were resuspended in 40% Percoll and layered on top of 70% Percoll. After centrifugation at room temperature for 25 minutes at 1135*g*, the cells in the interphase were harvested, washed with medium, and used for *i*NKT cell analysis. For *i*NKT cell enrichment from splenocytes, B cells were depleted using anti–mouse IgG magnetic beads. In brief, splenocytes were resuspended in HBSS supplemented with 10% FCS, and then mixed with anti–mouse IgG-conjugated BioMag beads (QIAGEN). After incubation for 40 minutes on a MACSmix Tube Rotator (Miltenyi Biotec) at 4°C, the beads were magnetically removed, and the nonbinding cells were harvested for further analysis.

### iNKT cell subset analysis by intracellular staining.

*i*NKT cells were first identified by PBS-57–loaded mouse CD1d tetramers followed by staining for surface markers, as previously described (48). For each staining, 5 million cells were fixed in 150 μL of a 1:3 mixture of concentrate/diluent working solution of Foxp3 transcription factor staining buffer (eBioscience) plus 100 μL FACS buffer (0.5% BSA, 0.1% sodium azide in HBSS), after which they were incubated at room temperature for 20 minutes. Cells were washed twice with 1× permeabilization buffer (eBioscience) before adding antibodies for transcription factors, such as PLZF, RORγt, and T-bet. After 1 hour of room temperature incubation, the cells were washed and analyzed by flow cytometry.

### Detection of intracellular cytokine production.

Freshly isolated thymocytes were stimulated with PMA (25 ng/mL) and ionomycin (1 μM) (both from Sigma-Aldrich) for a total of 5 hours. Brefeldin A (eBioscience) was added for the last 4 hours of incubation. Stimulation was terminated by washing cells in ice-cold FACS buffer. For dead-cell exclusion, stimulated cells were stained with Ghost Dye Violet 510 (Tonbo Biosciences) for 25 minutes at 4°C, and excess reagents were washed out with FACS buffer. Surface staining was performed before the cells were fixed and permeabilized using the Foxp3 transcription factor staining buffer kit according to the manufacturer’s instructions (eBioscience). Cells were incubated at room temperature for 1 hour after adding the anti-cytokine antibodies, i.e., IL-17 and IFN-γ. After incubation, stained cells were washed and analyzed by flow cytometry.

*In vitro stimulation of iNKT cells withα-GalCer*. Thymocytes were processed into single-cell suspension (2 × 10^6^ cells/mL) in RPMI-1640 media supplemented with 10% FCS and plated into 24-well plates with different concentrations of α-GalCer (30, 100, and 300 ng/mL). Cells were incubated overnight at 37°C in a 7.5% CO_2_ incubator before staining and analysis by flow cytometry. The α-GalCer (KRN7000, Funakoshi) solution was prepared as previously described (49).

### Intracellular staining of thymic iNKT cells.

For intracellular staining and analysis of thymocytes, dead cells were excluded by Ghost Dye Violet 510 (Tonbo Biosciences) staining. Cells were then stained with PBS-57–loaded mouse CD1d tetramers followed by staining for surface makers. Cells were fixed with IC fixation buffer (eBioscience) and permeabilized using Foxp3 transcription factor staining buffer kit (eBioscience) according to the manufacturer’s instructions. Cells were incubated for 1 hour at room temperature after adding the antibodies, such as anti-perforin and anti-granzyme A. After incubation, stained cells were washed and analyzed by flow cytometry.

### Statistics.

Results are shown as mean ± SEM. A 2-tailed Student’s *t* test was used to calculate *P* values. *P* values of less than 0.05 were considered significant. Statistical analyses were performed using GraphPad Prism 8 software.

### Study approval.

Animal experiments were approved by the National Cancer Institute Animal Care and Use Committee. All mice were cared for in accordance with NIH guidelines.

## Author contributions

SL, JK, and AC designed and performed the experiments, analyzed the data, and contributed to the writing of the manuscript. PWP provided reagents, reviewed the data, and commented on the manuscript. JHP conceived the project, analyzed the data, and wrote the manuscript.

## Supplementary Material

Supplemental data

## Version 1 09/22/2021

Electronic publication
