# Supplementary material for: CD138 expression is a molecular signature but not a developmental requirement for RORγt+ NKT17 cells
Source: JCI Insight. 2021 Sep 22;6(18):e148038. doi: 10.1172/jci.insight.148038 (PMC8492317; doi:10.1172/jci.insight.148038)
Supplement: Supplemental data [file jciinsight-6-148038-s093.pdf]

Supplementary Figure 1

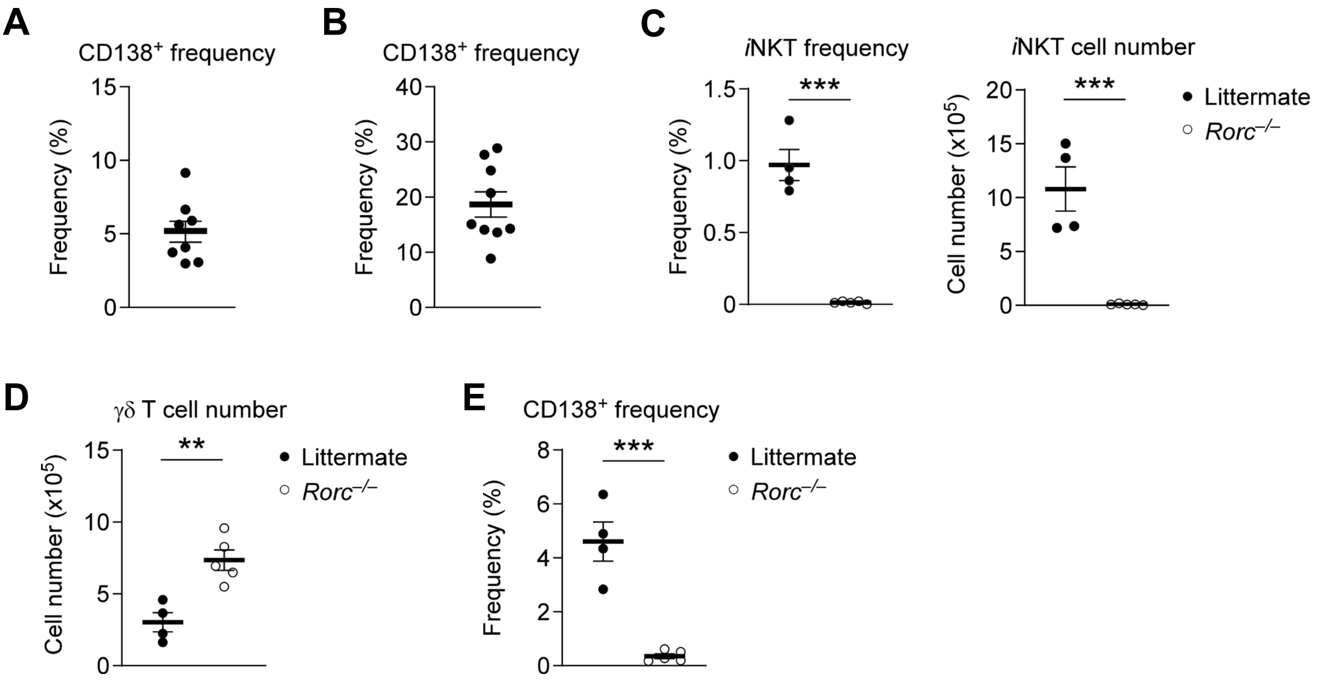

Supplementary Figure 1. CD138 expression in BALB/c thymocytes

(A) The graph shows the frequency of CD138^{+} γδ T cells among total thymic γδ T cells of BALB/c mice. Data are summary of 3 independent experiments with a total of 8 BALB/c mice.

(**B**)The graph shows the frequencies of CD138^{+} *i*NKT cells among total thymic *i*NKT cells of BALB/c mice. Data are summary of 4 independent experiments with a total of 9 BALB/c mice.

(C) The graphs show frequencies and cell numbers of *i*NKT cells in *Rorc*$^{-/-}$ and WT littermate BALB/c thymocytes. Results are the summary of 3 independent experiments with a total of 5 *Rorc*$^{-/-}$ and 4 WT littermate mice.

(D) Thymic γδ T cell numbers in *Rorc*$^{-/-}$ and WT littermate BALB/c mice. Results are summary of 3 independent experiments with a total of 5 *Rorc*$^{-/-}$ and 4 WT littermate mice.

(E) The graph shows the frequency of CD138^{+} γδ T cells among total thymic γδ T cells of *Rorc*$^{-/-}$ and WT littermate BALB/c mice. Results are summary of 3 independent experiments with a total of 5 *Rorc*$^{-/-}$ and 4 WT littermate mice.

Supplementary Figure 2

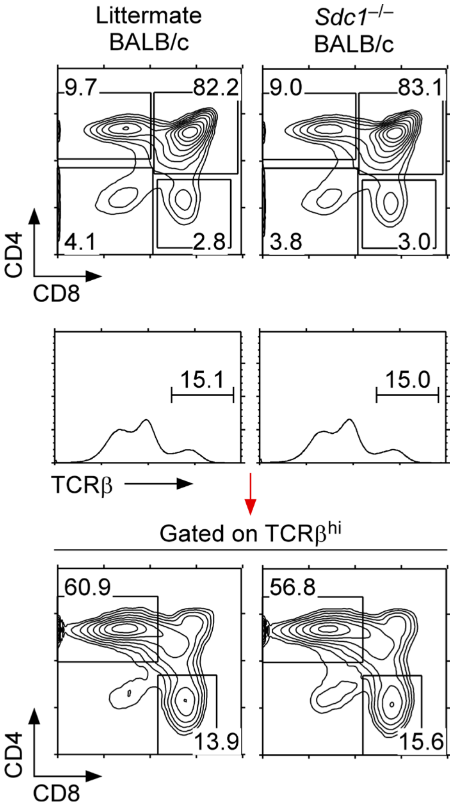

Supplementary Figure 2. T cell development in *Sdc1*$^{-/-}$ BALB/c mice

Contour plots show CD8 versus CD4 profiles of total (top) and TCRβ^{hi}-gated (bottom) *Sdc1*$^{-/-}$ and WT littermate BALB/c thymocytes. The results are representative of 6 independent experiments with a total of 10 *Sdc1*$^{-/-}$ and 10 WT littermate BALB/c mice.

Supplementary Figure 3

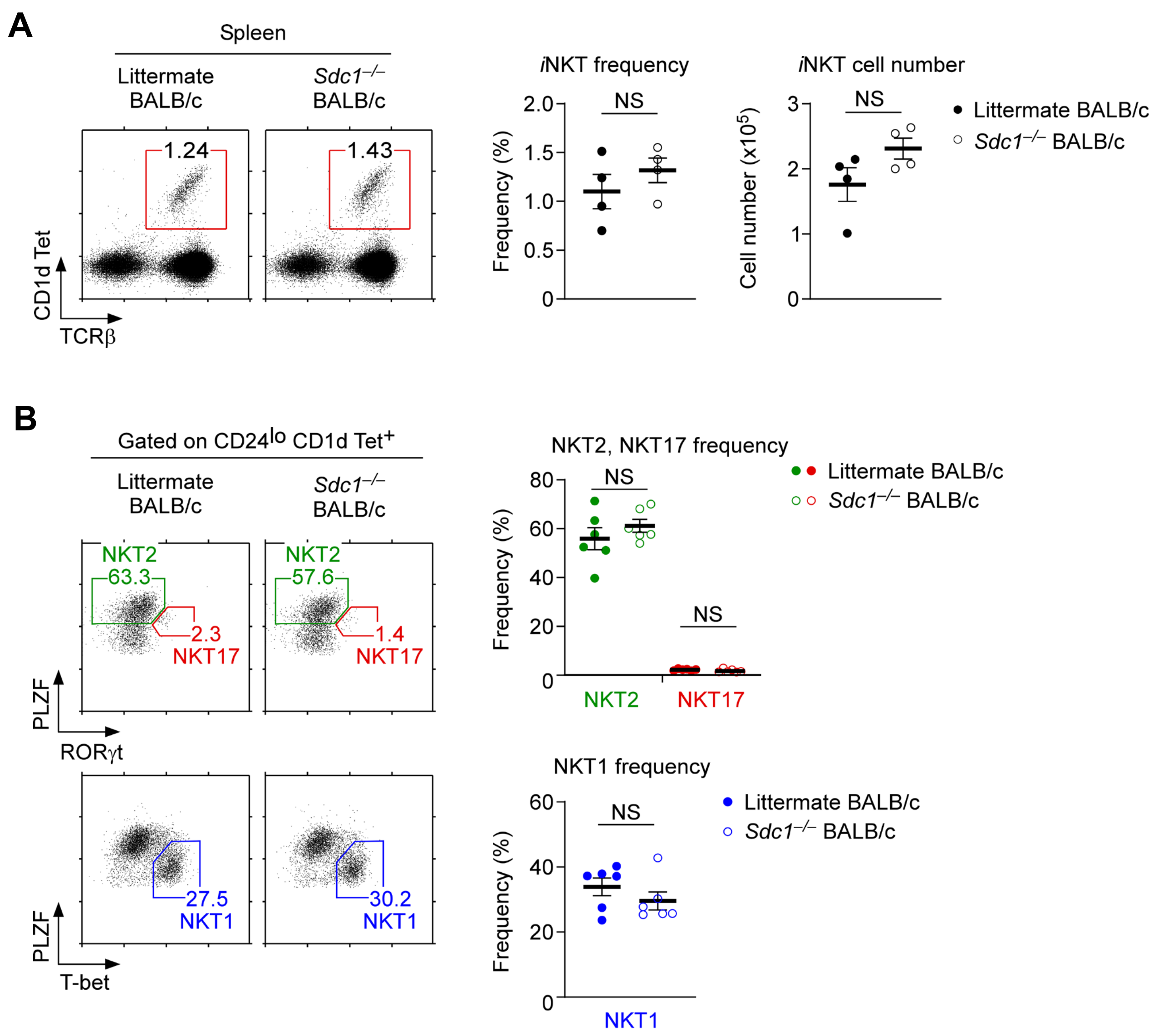

Supplementary Figure 3. Splenic *i*NKT cells in *Sdc1*$^{-/-}$ BALB/c mice

(A) Identification of *i*NKT cells among B cell-depleted splenocytes of *Sdc1*$^{-/-}$ and WT littermate BALB/c mice. The dot plots are representative (left), and the *i*NKT frequency and number graphs (right) show the summary of 2 independent experiments with a total of 4 *Sdc1*$^{-/-}$ and 4 WT littermate BALB/c mice. Numbers in the box show frequencies of *i*NKT cells among B cell-depleted splenocytes.

(B) Subset distribution in *Sdc1*$^{-/-}$ and WT littermate BALB/c splenic *i*NKT cells. The frequencies of NKT1, NKT2, and NKT17 cells were determined by T-bet versus PLZF and RORγt versus PLZF expression. The dot plots are representative (left), and the graphs show the summary (right) of 3 independent experiments with a total of 6 *Sdc1*$^{-/-}$ and 6 WT littermate BALB/c mice.

Supplementary Figure 4

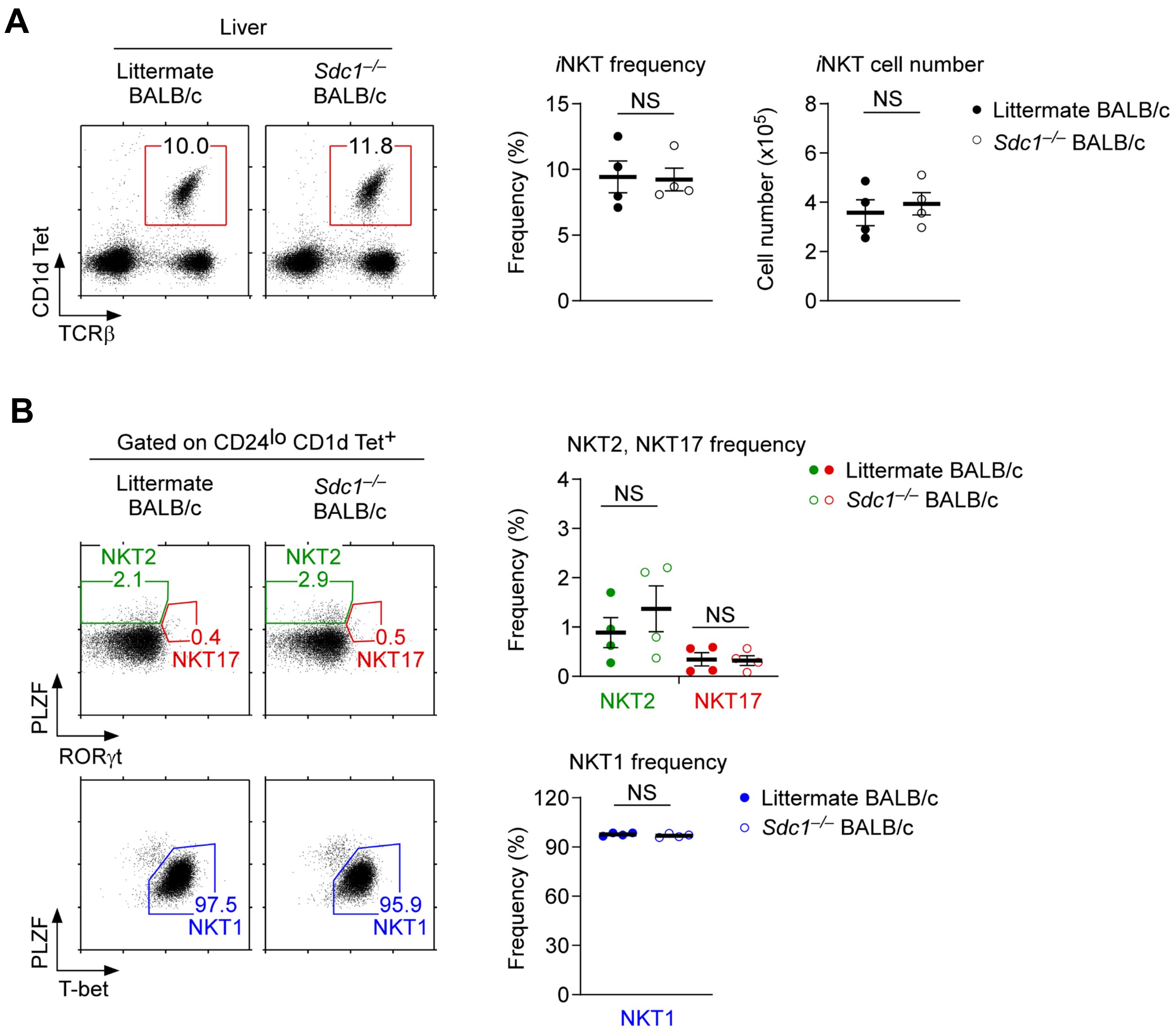

Supplementary Figure 4. Liver *i*NKT cells in *Sdc1*$^{-/-}$ BALB/c mice

(A) Identification of *i*NKT cells in the liver of *Sdc1*$^{-/-}$ and WT littermate BALB/c mice. The dot plots are representative (left), and the *i*NKT frequency and number graphs (right) show the summary of 2 independent experiments with a total of 4 *Sdc1*$^{-/-}$ and 4 WT littermate BALB/c mice. Numbers in the box show frequencies of *i*NKT cells among total CD45^{+} liver lymphocytes.

(B) *i*NKT subset distribution in *Sdc1*$^{-/-}$ and WT littermate BALB/c liver cells. The frequencies of NKT1, NKT2, and NKT17 cells were determined by T-bet versus PLZF and RORγt versus PLZF expression. The dot plots are representative (left), and the graphs show the summary (right) of 2 independent experiments with a total of 4 *Sdc1*$^{-/-}$ and 4 WT littermate BALB/c mice.

Supplementary Figure 5

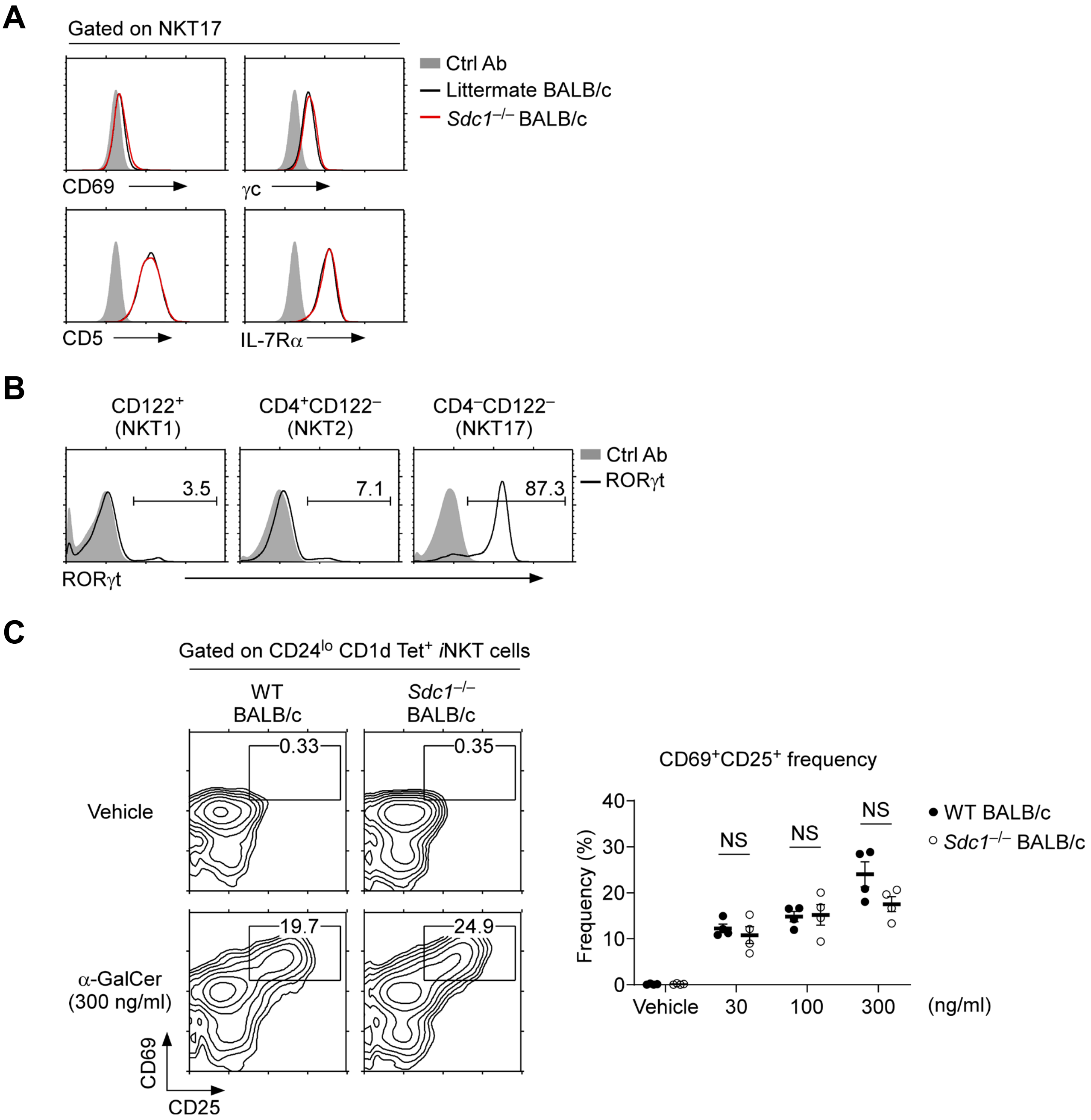

Supplementary Figure 5. Characterization of thymic *i*NKT subsets in *Sdc1*$^{-/-}$ BALB/c mice
(A) CD69, CD5, γc and IL-7Rα expression were assessed on NKT17 cells of *Sdc1*$^{-/-}$ and WT littermate BALB/c thymocytes. The histograms are representative of 2 independent experiments with a total of 2 *Sdc1*$^{-/-}$ and 2 WT littermate BALB/c mice.
(B) RORγt expression in thymic *i*NKT subsets as identified by CD122 and CD4 staining. The histograms are representative of 2 independent experiments with a total of 3 BALB/c mice.
(C) Surface CD69 and CD25 expression was assessed on mature *i*NKT cells of *Sdc1*$^{-/-}$ and WT littermate BALB/c thymocytes upon overnight *in vitro* stimulation with the indicated amounts of α-GalCer. Contour plots are representative, and the graph show the summary of 3 independent experiments. Results are summary of 3 independent experiments with a total of 4 *Sdc1*$^{-/-}$ and 4 WT BALB/c mice.

Supplementary Figure 6

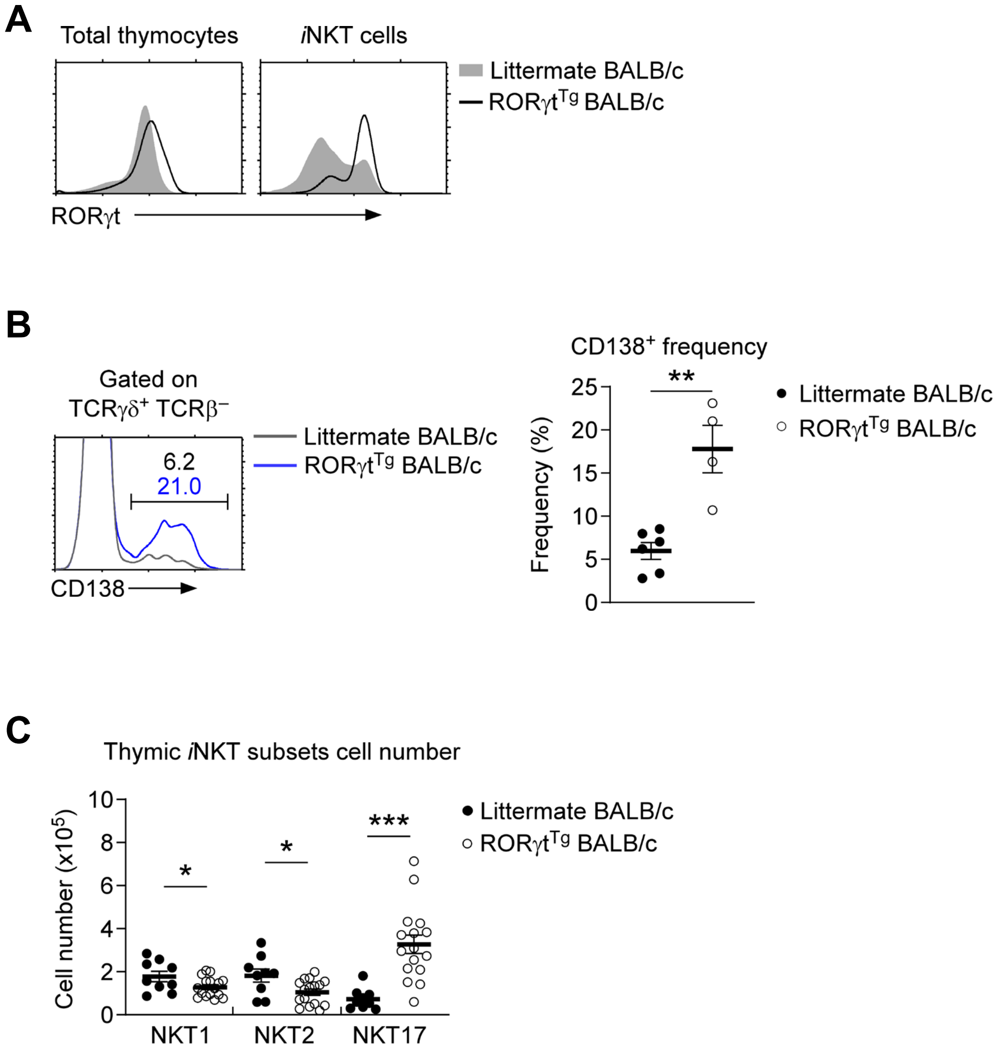

Supplementary Figure 6. *i*NKT and γδ T cell analysis in RORγt^{Tg} BALB/c thymocytes
(A) The intracellular RORγt contents in total thymocytes and total *i*NKT cells were assessed in RORγt^{Tg} and WT littermate BALB/c mice. The histograms are representative of 2 independent experiments with a total of 5 RORγt^{Tg} and 3 WT littermate BALB/c mice.
(B) CD138 expression on thymic γδ T cells of RORγt^{Tg} BALB/c thymocytes. The histogram (left) identifies CD138^{+} γδ T cells, and the graph (right) summarizes the frequency of CD138^{+} γδ T cells. The results are representative of 2 independent experiments with a total of 4 RORγt^{Tg} and 6 littermate WT BALB/c mice.
(C) Thymic NKT1, NKT2 and NKT17 cell numbers were determined in RORγt^{Tg} and WT littermate BALB/c mice. The results show summary of 4 independent experiments with a total of 16 RORγt^{Tg} and 9 WT littermate BALB/c mice.

Supplementary Figure 7

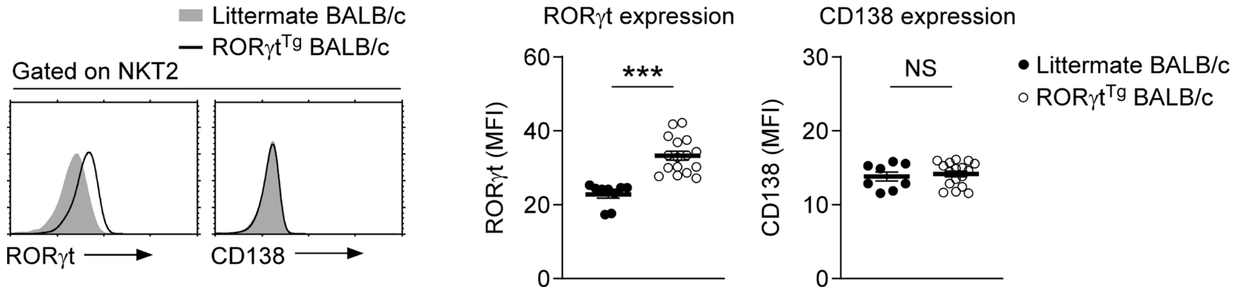

Supplementary Figure 7. RORγt and CD138 expression in NKT2 cells of RORγt^{Tg} BALB/c thymocytes

The histograms show RORγt and CD138 expression in thymic NKT2 cells (left). The graphs show the MFI of RORγt and CD138 in NKT2 cells of the indicated mice (right). The results show summary of 4 independent experiments with a total of 16 RORγt^{Tg} and 9 WT littermate BALB/c mice.

Supplementary Figure 8

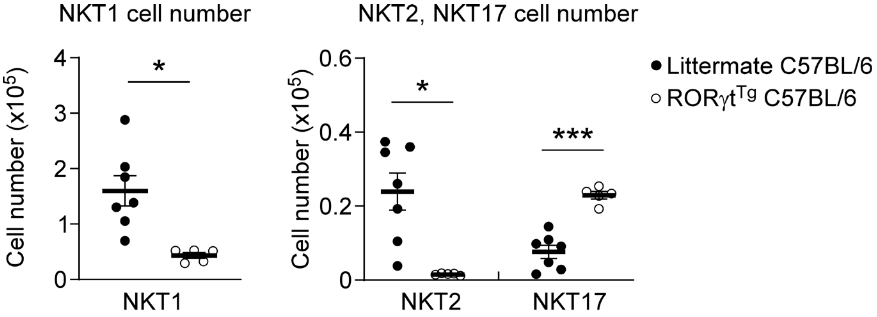

Supplementary Figure 8. Characterization of thymic *i*NKT subsets in RORγt^{Tg} C57BL/6 mice Thymic NKT1, NKT2 and NKT17 cell numbers were determined in RORγt^{Tg} and WT littermate C57BL/6 mice. The results show summary of 3 independent experiments with a total of 5 RORγt^{Tg} and 7 WT littermate C57BL/6 mice.
